# Military societies: self-governance and criminal justice in Indian country

**DOI:** 10.1007/s11127-022-01004-1

**Published:** 2022-10-21

**Authors:** Adam Crepelle, Tate Fegley, Ilia Murtazashvili

**Affiliations:** 1 George Mason University, Fairfax, USA; 2 University of Pittsburgh, Pittsburgh, USA

**Keywords:** Criminal justice, Self-governance, Traditional institutions, B52, H12, H71, K14

## Abstract

We argue that criminal justice institutions must be accessible to citizens, legitimate and have capacity to enforce law. Such was the case with the military societies of the Plains Indians: a system of criminal justice that predated the time of European contact and which remained a significant source of law and order in Indian country until the Indian Wars concluded at the end of the nineteenth century. Nonetheless, the federal government attempted to replace military societies with federal police starting circa 1850. Despite such attempts, we show that military societies remain an important institution for criminal justice on the contemporary Northern Cheyenne Reservation. When the federal government shirked on policing during the coronavirus pandemic, military societies took over important policing functions. This does not mean that traditional military societies should replace federal enforcement; rather, it shows that until the quality of federal policing improves, traditional institutions of criminal justice remain an important source of public safety in Indian country.

This is effectively a lawless land. People know that there’s no consequences. Crimes that were taboo are now normal and they feel like some kind of invisibility for them. That needs to change (Waylon Rogers a member of the Northern Cheyenne Tribal Council Quoted in Hamby, 2020)

## Introduction

In addition to facing a deadly pandemic, the Northern Cheyenne Reservation confronted increasing crime during the coronavirus pandemic shutdowns of 2020. One likely reason for the increase in crime was that the federal government decided to stop policing the reservation during the pandemic even though it is treaty bound to provide public safety on the Northern Cheyenne Reservation. The reason why the federal government’s shirking presented a challenge for public safety is because the Northern Cheyenne, as well as every other tribe, lack criminal jurisdiction over non-Indians, and the maximum penalty most tribes can impose is a single year in jail.^1^ Thus, tribes depend on federal law enforcement, and the federal government’s shirking created a major enforcement challenge. In response, the tribe filed a lawsuit that the United States breached its fiduciary duty. The crime resulting from the lack of law enforcement was one reason why a member of the tribe’s council referred to the reservation as a “lawless land.” But the tribe did more than file a lawsuit. They authorized their military societies, a traditional institution of criminal justice, to help in the administration of criminal justice. According to the accounts we found, they did an effective job providing policing services.

Those who trust the federal government might be surprised that it shirked on its responsibilities during hard times; those unfamiliar with traditional governance in Indian country might not have expected that military societies—the traditional system of criminal justice in Indian country—could step up to provide policing services. We argue here that this is not so surprising from a public choice perspective. In fact, public choice is especially well suited to explain several key features of policing in Indian country, including why military societies were effective as an institution of criminal justice from the time before European contact through the end of the Indian Wars (which were mostly concluded by 1900); why the federal government nonetheless attempted to replace traditional criminal justice institutions among all Indians starting circa 1850; and why military societies are still able to provide policing services on contemporary reservations.

Military societies, in the most general sense, are traditional institutions of criminal justice that were appointed by and operated in the system of tribal government in Indian country. Military societies predated European contact and remained an important institution until the end of the Indian Wars. Our focus is on the military societies of the Plains Indians: those Indians whose traditional homelands are located in present-day Minnesota, the Dakotas, Iowa, Kansas, Nebraska, Oklahoma, Montana, and Wyoming. The reason why we focus on their military societies is that while traditional policing institutions and military societies were common among most tribes, these societies are most closely associated with Indians in the Plains. Pragmatically, anthropological accounts have focused most extensively on the history and structure of these societies and so these are also the societies for which we have the most available information.

Our interest is in comparing traditional enforcement with government enforcement. By traditional enforcement, we mean enforcement by tribal nations; government enforcement refers primarily to federal enforcement by the Bureau of Indian Affairs (BIA). Our theory is that criminal justice institutions must satisfy three conditions: accessibility to citizens, legitimacy, and capacity to enforce laws. We then show that military societies satisfied these conditions historically and that they continue to on the Northern Cheyenne Reservation despite being financed by voluntary contributions. Policing by the BIA, in contrast, has typically not satisfied these conditions, either historically or on contemporary reservations; the high levels of crime on contemporary reservations are evidence of enforcement failure by the BIA.

Public choice also provides insight into why the federal government attempted to replace an arguably efficient institution (military societies) with an inefficient institution (policing by the federal government) starting circa 1850. Previous public choice analysis of Indian-white relations has focused on the role of the federal government’s military capacity as an explanation for federal Indian policy in the nineteenth century. As Anderson and McChesney (1994) showed, treaties among the federal government and Indian tribes were relatively common in the early nineteenth century, but by the middle of that century and especially after the Civil War concluded in 1865, the federal government relied almost exclusively on the Army to solve conflicts arising with Indian Nations. We argue that one of the implications of Anderson and McChesney’s (1994) analysis is that the federal government also had few incentives to respect indigenous tribal institutions once the reservation system was established; indeed, the federal government used reservations as a mechanism to destroy indigenous cultures and compel assimilation.

Our analysis also highlights the costs of imposing foreign policing institutions on reservations. One of the themes in public choice analysis of economic development is that foreign-imposed exogenous institutions are often inferior to indigenous institutions that are formalized (Boettke et al., 2008). Anderson and Parker (2022) argue similarly: federal rules which constrain autonomy of social structures based on customs and culture contributed to economic underdevelopment on reservations. The political economy of policing in Indian country illustrates the costs of such foreign imposition of institutions on criminal justice.

Our paper is organized as follows. In Sect. 2, we develop a theory of criminal justice institutions that enables us to compare federal government enforcement of law and order with enforcement by military societies. Section 3 considers the gradual replacement of an arguably efficient institution of criminal justice (military societies) with an arguably inefficient one (federal policing on reservations) starting circa 1850. Section 4 is a case study of policing on the Northern Cheyenne Reservation during the pandemic that shows the ongoing significance and effectiveness of military societies as a self-governing institution of criminal justice. Section 5 discusses several extensions of our analysis including the role of military societies in co-production of policing services. Section 6 concludes.

## Public choice and criminal justice

A rich tradition in public choice considers criminal justice institutions. One of the themes in this literature is that the government is not the only source of law and order and that governments often shirk on their obligations to provide policing services. The first point—that government does not have a monopoly on law and order—reflects the more general finding that public goods are often provided privately (Candela & Geloso, 2018; Clark & Powell, 2019). Some of this literature’s results are based on “folk theorems,” which in game theory explain how individuals can cooperate without relying on a third party to enforce their agreements. Hadfield and Weingast (2012) note that compliance with what they call “law” can be voluntary (individuals punish themselves for wrongful conduct); individuals can punish one another (as in repeated games); or the rules can be enforced collectively (members of a group can collectively refuse to deal with someone who has done wrong). However, complications arise when coercion is present. Cowen and Sutter (2005) argued that whenever competing organizations attempt to provide law, they end up fighting. For example, it is in the interest of both roving bandits and the general population to exit the Hobbesian jungle, and bandits will compete or collude to establish a government, ending up as stationary bandits (Olson, 1993). As Leeson (2007, p. 468) explains, folk theorems “cannot prevent ‘violent theft’ such as extortion, expropriation, or other kinds of plunder for which the predator’s superlative strength allows him to physically overwhelm the victim.” Rather, what is necessary is typically to understand the ways in which rules shape and constrain incentives of self-governing criminal justice institutions.

To address the challenges with folk theorems, it is necessary to specify the conditions under which self-governance of policing works. Coyne and Leeson (2012) did this in comparing traditional enforcement with government enforcement. They argued that effective criminal justice must be accessible to citizens, must incentivize judicial administrators to pursue justice instead of private ends, and must generate useful information about the accused criminals’ guilt or innocence.

In what follows, we also argue that criminal justice institutions must satisfy several conditions, though our conditions differ. Perhaps most significantly, we have as a condition enforcement capacity. Given Coyne and Leeson’s emphasis on the “truth” of traditional methods of adjudication of guilt or innocence, it makes sense for them to ignore enforcement; but because we are interested in the implementation of criminal justice, we make enforcement capacity an explicit condition for effective criminal justice. In addition, we do not consider explicitly the incentives for military societies to ascertain guilt or innocence of individuals, which we leave for future consideration.

First, criminal justice institutions must satisfy the requirement of accountability to citizens. We view access as significant (as Coyne and Leeson do), though in a more general sense, the first condition for criminal justice must be accountable to citizens. Access is one way to ensure accountability; but, following Elinor Ostrom’s insight into policing, it is necessary that those entrusted to police are accountable to provide criminal justice (Boettke et al., 2016). The second condition, we argue, is legitimacy. Criminal justice has a significant cost of compliance with rules, and so the ability to provide criminal justice is likely to depend on how legitimate the enforcers are perceived to be (Tyler, 2003). This condition is implicit in Coyne and Leeson’s framework, but in comparisons of traditional with government policing, it cannot be assumed that one form (traditional versus government) is legitimate. Third, it is necessary to have capacity to enforce laws. Absent capacity, enforcing law will be challenging, if not impossible, though as we show, capacity can include willingness of people to volunteer labor; indeed, the military societies on the contemporary Northern Cheyenne Reservation volunteered their labor and had few resources, yet were able to contribute meaningfully to public safety.

Our hypothesis is that either military societies or the federal government will be able to provide policing services provided they satisfy the conditions above. In the remainder of the paper, we consider the extent to which military societies and federal policing satisfied these conditions; we also relate each system to provision of criminal justice in Indian country.

## The transition from enforcement by military societies to the federal government

The conventional origin of policing departments organized at the municipal level, with officers employed by government, is the establishment of the London Metropolitan Police Department in 1829. Different rationales are given for consolidation: Benson (1994) argues that statist policing was not a consequence of efficiency considerations, while Allen and Barzel (2011) contend that consolidation resulted from technical innovations during the Industrial Revolution.

These debates about the evolution of policing inform our analysis of Indian country, which also offers insight into the veracity of the contrasting accounts. In Indian country, the changes in policing are less accurately described as consolidation (scaling up of local departments) but were rather the centralization of policing and replacement of local institutions of criminal justice. The development of policing institutions in Indian country is more consistent with a statist explanation, mainly because the federal government attempted to eliminate traditional tribal policing institutions, including military societies, even though they were effective sources of criminal justice. In what follows, we show that military societies were efficient and that federal actions are best thought of as imposition of foreign, exogenous, and inefficient institutions.

### Military societies of the plains Indians

Military societies are associated most commonly with Plains Indians, although we discuss alternative systems of traditional policing as well. In addition, as we noted in the Introduction, anthropological accounts have focused on the Plains Indians’ military societies. The anthropological studies find that among Plains Indians, military societies essentially were universal, and so the account that follows is a general description of these societies.

Humphrey (1942) analyzed data from 32 distinct tribes in the Plains regions that established “warrior” or “police” societies, with a focus on the Blackfoot, Crow, Cheyenne, and Kiowa tribes (not enough data were available on the Comanche to include in his study), along with Southern Plains tribes, and a village in the upper Missouri River’s reaches (Meadows 2002 reported information on the Comanche that aligns with earlier research). Humphrey’s emphasis was on the ways military societies were organized from the time of European contact through the mid-nineteenth century, though his interview accounts provided special insight into the nature and function of these societies circa 1850. We can summarize Humphrey’s analysis of the organization of tribes, and the role of military societies within tribes, as follows.

Each tribe had an executive, usually a chief, along with a tribal council that informed the decisions of chiefs. The extent to which these councils constrained the chiefs varied among the Plains Indians but what was universal is that military societies operated within the system of tribal government. They were not part of the tribal council, nor did they typically include chiefs. The responsibility of these societies was to enforce rules agreed upon by chiefs and councils, most of which were the result of a deliberative process. Military societies were institutions in that they were governed by specific rules. In all tribes, military societies were subordinate to the will of tribal councils. In some tribes, the councils had formal authority to veto military society proposals, such as among the Crow. Members of military societies were typically appointed, with definitive tenures that varied (for a year, a season and, for some tribes, a day) (Humphrey, 1942, pp. 147–149).

The types of offenses that military societies policed could be divided broadly into offenses against tribal goods and those against the units of the group (Humphrey, 1942). An example of the type of policing performed by military societies is policing the buffalo hunt: hunting buffalo on foot with a spear or bow is exceedingly difficult for an individual; thus, group hunts were far more efficient. Group hunting requires positioning hunters and timing their movements—a single hunter charging too soon could scatter the entire herd of buffalo. A leadership system is required to coordinate hunting party behavior. While buffalo hunting became much easier after the acquisitions of the rifle and horse, Plains Indians continued their group hunts, and similar organizational concerns prevailed. As a result, governance structures emerged and were quite strong: as Humphrey (1942, p. 150) puts it, “complete, almost inexorable control over the population of a camp was exercised primarily during the period of the communal buffalo hunt.”

As Meadows (2002) explains, among the Plains Indians, before circa 1850, military societies specialized in mobile plains hunting and gathering, including policing large communal hunts (by confiscating illegally seized game, destroying property, and corporal punishment), as well as coming together for social or sacred gatherings. Duties were performed by a particular society or clan, by temporarily appointed members of a society, by societies in turn, or by groups without associational affiliations, such as clans or honorary classes of braves. The societies provided services (including charity) to the larger community, supplied camp security and specified its layout, collectively fostered military ideology, and often asserted supernatural powers. Military ideology was maintained and promoted at the pan-tribal level through rituals, singing, and coup recitation (recounting successes, or coups, in conflicts) as well as redistributive giving away of property.^2^ (See Johnsen, 2022 on the economic and political rationale for gift giving among Indian tribes in the Pacific Northwest historically.)

Meadows (2002) summarizes the organizational features from the time of European contact to circa 1850 as follows: voluntary, cross-band membership; definitive rules for participation (age-graded or non-age-graded were the two basic rules for membership); names associated with dress, dance, duty, or associated group ideology; and proper behavior in small group meetings or larger tribal assemblies. Rising warfare on the Great Plains led to emphasis of more warlike postures, with many accounts written by whites describing all Plains Indian military societies as Dog Soldiers. Although not all military societies were called Dog Soldiers and they did much more than prepare for war, military societies shared a warrior ideology. Their functions encompassed matters of war and ceremonial hunts.

Military societies often relied on superstition and it appears to have conferred greater legitimacy on them. The Cheyenne’s Dog Society, for example, enforced banishments (the penalty for wrongful deaths), justifying the need for penalties based on superstition:

Since the death of a Cheyenne at the hands of a tribesman “bloodied” the arrows of the Medicine Arrows and the Medicine Hat (upon whom tribal welfare rested), resulting in game shunning the country and the failure of war parties, it was necessary to discover the culprit (Humphrey, 1942, p. 157).

In the case of the Cheyenne, the supernaturally caused consequences of the crime (scarcity of game, failure in war) made it, in the eyes of the Cheyenne, an offense against the material welfare of the tribe. This aligns with Leeson and Suarez’s (2015) argument that superstitions can improve self-governing collective action: in the case of military societies, superstition increased incentives to obey rules, as well as added legitimacy to their enforcement by military societies.

Military societies were not the only organizations adopted by the Plains Indians. The anthropological accounts mention “shield societies,” which were smaller, focused on shield and warfare power, and more secretive.

Warriors from specific tribes also were relied on for specific purposes. The most significant, cross-cutting organization for policing was the military society among the Plains Indians. It appears to have been a general feature circa 1850 as well as after, as the military societies continued to function as skirmishes and battles with US soldiers increased, especially as settlers began to increase in larger numbers circa 1859 (the year of discovery of gold and silver in Colorado). We discuss dynamics from 1850 until the Northern Cheyenne were forced to reservations in Montana by circa 1880.

### Federalization of policing in Indian country

Every major US city had a municipal police department by 1900. Part of the reason was the progressive ideology that government should play a larger role in society (Holcombe, 2019). Progressive public administrators tended to believe that centralization would improve the quality of public goods provision, including policing services (Aligica et al., 2019).

The reservation system was not specifically based on progressive ideology, but it shared the belief that centralized government control over all aspects of reservation life was best for the Indians. Accordingly, criminal justice institutions became increasingly centralized. But Indians who were on reservations already were adapting their own policing institutions as the federal government began imposing new policing institutions. This was perhaps most clear among tribes from the East and Southeast that were among the first to be forced to reservations in the 1830s. For example, the Five Civilized Tribes—Cherokee, Choctaws, Muskogee, Chickasaws and Seminole, all initially located in the East and Southeast—included codes of personal responsibility, extended to clans, who were expected to punish members for transgressions (a substantial literature considers such norms in other contexts, e.g., Greif, 2006 on collective reputations in modern commercial law and Umbeck’s, 1977 analysis of personal reputations for violence as a source of order during the Gold Rush in California circa 1849–1850). Each of the tribes gradually replaced its laws of clan revenge with codified policing institutions circa the 1860s (Karr 1998). Such codification included emergence of Lighthorsemen, elected law enforcers and adjudicators who combined the roles of sheriff, judge, jury, and executioner (Blackburn, 1980). The Cherokee Lighthorse police force handled petty crimes and more serious violations, including horse theft, with its jurisdiction later expanding to major crimes (robbery, murder, and rape) along with disruptions of the public order, including public intoxication. Part of the reason was functional: growing populations, intermarriages among Indians, and interactions between Indians and non-Indians in Indian country benefitted from more standardized enforcement.

Some tribes were given partial autonomy over criminal justice. After the Long Walk—the violent deportation of Navajo from Arizona to New Mexico from 1864 to 1866—the federal government initially proposed some semblance of local policing. Specifically, the government asked war chief Manuelito to recruit 100 Navajo warriors to catch Navajo accused of raiding livestock. The Navajo police were perhaps too effective, for crime declined so much that they were disbanded in 1873 (after only a year of operation); when the Navajo police were reinstated in 1874, they were paid out of tribal annuities rather than with additional funding from the federal government (Jones 1966). Although they were reinstated after a year, they lost funding, and hence were less able to provide policing services.

Since the federal government largely shifted to a strategy of warfare in the Plains, there was not much opportunity for partnerships with the tribes and federal government over policing. Rather, the dynamic was largely one of military societies asserting increasingly military roles in self-defense. The Army was increasingly used to provide security for settlers along the various trails (trading routes) linking East and West. Many Plains Indians signed treaties from 1850 through the end of the Civil War, but during that time, many bands, led by their military societies, resisted the government. In 1878, Congress approved establishing the federal Indian police and, by 1890, federal police were assigned to nearly all reservations. It was not tribal policing, but federal policing: the new Indian police forces received allotments for themselves and their families from the federal government, who was their employer. Federal employment regulations required them to be “civilized” (hard working and abstaining from alcohol), wear uniforms, curtail the tribal chiefs’ prerogatives, and enforce non-Indian law, for which they earned better pay and were armed with revolvers. Decisions about budgets and staffing were made by the federal government (Hagan, 1966). Thus, the federal policy on all reservations was for Washington to hire tribal police and judges, which they did by adopting a divide-and-conquer strategy whereby they employed Indians from one tribe to police other tribes. For example, war chiefs Sitting Bull and Crazy Horse, leaders fighting for self-determination, were killed by BIA police. In addition, the federal government attempted to ensure compliance by withholding annuities to tribes who failed to live up to BIA expectations, including with respect to policing (Hagan, 1966).

Federal control of reservation rapidly accelerated in the wake of the Supreme Court’s 1883 decision in *Ex Parte Crow Dog* which held that the United States lacked authority to punish Indian on Indian crimes committed on a reservation. Two years after the *Ex Parte Crow Dog* decision, Congress passed the Major Crimes Act (MCA). Members of Congress who supported the legislation were upset that the Sioux resolution of the killing—restitution—was too lenient even though for centuries, restitution was the only punishment meted out for crimes committed under the common law. The MCA was based on the rationale that tribes were not capable of punishing “major crimes.” Although it acknowledged that Congress lacked constitutional authority to pass the MCA, the Supreme Court affirmed the MCA in *United States v. Kagama* (1886) because the Indians “are the wards of the nation.” Additional efforts to eliminate tribal sovereignty over policing soon followed, including creation of Courts of Indian Offenses during the 1880s, further diminishing tribal authority over crime on reservations. A cumulative consequence was that tribes, which were capable of policing themselves, were forced into a centralized policing regime. As a consequence, when the Indian Wars ended, the federal government already had in place a clear process for imposing new, foreign institutions on reservations; there was little to no opportunity for policing to formally reflect traditional institutions.

Though the federal government asserted authority over reservations, shirking increased gradually. The number of funded Indian police dropped from 900 in 1880, to only 45 by the 1950s (Luna-Firebaugh, 2007). The lack of police officers led to accusations of reservations being “lawless.” Congress responded to that allegation in 1953 with Public Law 280, which granted the surrounding state policing authority over tribal lands. States were not provided with funding to police reservations, and many states had hostile relationships with tribes. Thus, states often shirked their reservation policing obligations (Crepelle, 2016). The central dynamic of criminal justice was a steady replacement of indigenous criminal justice institutions with centralized policing, initially the federal government and later the state governments.

## Society policing on contemporary reservations: the Northern Cheyenne case

The Cheyenne people initially lived in what is now Minnesota prior to contact with Europeans. Today, the Cheyenne are split into the Northern Cheyenne in Montana and the Southern Cheyenne and Arapaho Tribes in Oklahoma. The initial resettlement reflected tribal rivalries: they were first pushed to the Black Hills of South Dakota and Powder River Country in present-day Montana and Wyoming, later aligning with the Arapaho to push the Kiowa to the Southern Plains before the Lakota pushed them further west. Conflict with their traditional enemies, the Crow, and later with the US Army, led to a split in the bands in the mid-nineteenth century and to additional movements of the Cheyenne, further dividing the tribe.

To understand the background, we mention some of the key developments since they inform any understanding of the current situation on reservations. The Fort Laramie Treaty of 1851 assigned tribes of the Northern Plains to different lands to reduce intertribal warfare, with tribes receiving payments from the federal government to go along with the deal. The federal government’s interest was in securing trails, especially the Santa Fe Trail and Emigrant Trail, through Indian country. The lands promised to the tribes initially included areas of Colorado, Wyoming, Nebraska, and Kansas. By 1857, however, the US Cavalry was attacking Indians, leading the Cheyenne and other bands to retaliate. Within a few years, the Army had subjugated the tribes. The Army regional heads then proceeded to redistribute annuities to the Arapaho, leading the Cheyenne to initially move into Kiowa and Comanche country before they returned to their country in northern Colorado above the Southern Platte River.

The Colorado Gold Rush, which began in 1859, led to more settlers on the land of Plains Indians—there was peace for years between Indians and settlers, though the Cheyenne and Arapaho fought with the mountain Utes. Cheyenne continued to move where the buffalo were. The Colorado War in 1864 resulted from the Colorado Militia (a volunteer force) attacking Indians, leading to defensive raids and ultimately the Army’s intervention to close supply roads in 1864. Cheyenne Chief Black Kettle continued to push for peace, though there was raiding by Indians in response to aggression and several battles with hundreds to thousands of warriors involved circa 1865.

In 1868, George Armstrong Custer led Army troops to Black Kettle’s band despite the band being on a defined reservation; this led to the massacre on Washita River and upwards of one hundred Indians killed. In 1876, the Battle of Little Bighorn took place, with a war party of around 10,000 Indians from the Cheyenne, Lakota, and Arapaho bands killing Custer and much of the 7th Cavalry contingent. The US Army increased efforts to capture Cheyenne. In 1877, Lakota war chief Crazy Horse surrendered, and by 1879, Cheyenne bands were beginning to surrender, with some moving in with Sioux according to the 1868 treaty of Fort Laramie. In 1876, the US Army initiated a battle after which the Northern Cheyenne surrendered. Under pressure from the US government, the Northern Cheyenne agreed to go South, despite wanting to remain with the Sioux in the North. Circa 1878, the Northern Cheyenne, seeing inadequate rations, diseases such as malaria, and almost no buffalo to hunt, began a trek back to the north, fighting battles with US Army soldiers along the way. The Army eventually captured the Cheyenne, imprisoned, starved, and killed most of whom escaped. Eventually, the US government forced the Northern Cheyenne to a reservation in southern Montana, with some of the Cheyenne working as army scouts. The Northern and Southern Cheyenne were divided during the Indian Wars. After the Black Hills War (when Northern Cheyenne allied with the Lakota in 1876–1877) and the Sand Creek and Washita massacres, the Northern Cheyenne were relocated to Oklahoma. Although the Cheyenne were traditionally farmers before diffusion of horses, Northerners were unable to adapt because they were forced into agriculture despite having shifted to a predominantly hunting-based culture. The 1878 Northern Cheyenne Exodus involved a small band heading north, initially settling in Fort Keogh, Montana, before migrating south, closer to their current location. Currently, the Northern Cheyenne Tribe of the Northern Cheyenne Indian Reservation is a federally recognized tribe located in southeastern Montana. Approximately 5000 Cheyenne people live on the reservation, which is approximately 690 square miles. Its government headquarters is located in Lame Deer, Montana. The Cheyenne lands are bordered by the Crow reservation to the west and the Tongue River to the east.

### Government failure: BIA policing on the Northern Cheyenne Reservation

The BIA has formal authority to police the Northern Cheyenne Reservation. It has not worked well in either addressing policing issues or providing jails. Like many reservations, crime, including drug use, domestic abuse, and violence against Indian women, is an issue on the Northern Cheyenne Reservation. However, effectively no BIA cops are assigned to the reservation. While Rosebud County sheriff’s deputies maintain a presence, their role is mostly limited to traffic issues because they had no authority over crimes involving Indians until June of 2022.^3^ Some deputies signal trust by wearing a pin certifying their cultural sensitivity training. The Northern Cheyenne and Rosebud County have a good relationship, although there is political instability among the Northern Cheyenne: recently, the president was voted out of office after concerns were raised about corruption and lack of transparency, and turnover amongst council leadership is quite high (Associated Press 2022).

Even prior to the coronavirus pandemic, issues with BIA policing were evident. The BIA’s regulations suggest that 19 officers should have been assigned to the Northern Cheyenne Reservation. However, in the years prior to the pandemic, only six were present on average; often, only one officer was on duty (Aadland, 2020). Those are staggeringly small numbers of police for such a large area, especially compared to many municipal police departments, including those of small and medium-sized towns (Surprenant & Brennan, 2019).

The Northern Cheyenne were one of the first tribes to shut down during the pandemic, in part because they live in a rural area with few resources. Roadblocks were erected, which was easy since the reservation’s main roads all go through Lame Deer. However, with few police officers in place, the tribal government required assistance in maintaining the pandemic quarantine. Traditional military societies provided roadblock security, recruiting community members as unpaid volunteers.

The reason for the Northern Cheyenne’s reliance on military societies has to do with shirking by the BIA, which has proven unresponsive to tribal demands to enforce law on the reservation even before the pandemic. The social media pages of the Northern Cheyenne Tribe contain a series of press releases and correspondence between the tribe and the federal government, including congressional representatives, the Department of the Interior, and the Department of Justice, as well as between Congress and the executive branch, regarding the crisis in public safety on the Northern Cheyenne Reservation. US Senator Jon Tester of Montana has written several letters on behalf of the tribe. A letter dated November 20, 2019, to FBI Director Christopher Wray asked what he was doing with the $161 million increase in the FBI’s budget, which Sen. Tester had sponsored in fiscal year 2019 to improve safety in Indian country (Tester, 2019).

In a July 24, 2020, letter, the tribe reached out to Senators Steve Daines and Jon Tester and Congressman Greg Gianforte, requesting assistance in holding federal agencies accountable. “In the strongest possible terms, I plead for your help to address a public safety crisis on our Reservation which is the direct result of years of inexcusable neglect by the Bureau of Indian Affairs,” wrote then Northern Cheyenne President Rynalea Whiteman Pena (2020). The Northern Cheyenne highlighted the lack of resources provided by the US Department of Interior and the BIA’s Office of Justice Services (OJS). In the letter, Pena identifies four areas in which the bureau was failing the tribe: severely understaffed police, the closure of the local jail, lack of information sharing, and the agency’s absence, leading to rising crime and vigilantism. The Northern Cheyenne leader says that tribal citizens had lost faith in the BIA and that when a 911 emergency call is placed, the delay in a law enforcement response is too long, if police show up at all. “Our Reservation is a lawless society,” Pena wrote to the senators. “Drugs and alcohol are used openly and tearing apart our community. Violence has become commonplace.”

The letter emphasized a recent string of deaths that had gone uninvestigated by the BIA. In response, on August 12, Senator Tester (2020a) wrote a letter to Assistant Director of Indian Affairs Tara Sweeney and FBI Director Christopher Wray, mentioning the deaths of Lonnie Flatness, a retired Marine who was murdered in her home; Christy Woodenthigh, a mother of three who was run over and killed; and Kamani Littlebird, who was found hanging by the neck under suspicious circumstances. On August 12, Senator Steve Daines (2020) also wrote to Attorney General William Barr and Secretary of the Interior David Bernhardt, noting the death the previous day of Cory Blackwolf: “Cory was murdered in broad daylight, and his body taken away by his killers and put into a vehicle that sped away. These heinous acts and injustices cannot continue to happen and it is imperative that additional resources are promptly made available and you actively coordinate with the Tribe to address the crisis.”

Frustrated with the dearth of public safety on the reservation, on July 20, 2020, the tribe sent a letter to the BIA stating its intention to apply for a PL 638 contract permitting it to assume criminal investigation and drug enforcement duties. The tribe submitted its application on August 19, which was denied by the BIA on November 17 (Aadland, 2020). On December 15, the tribe filed a lawsuit against the BIA for failure to ensure public safety and improperly denying their application (Northern Cheyenne Tribe, 2020).

As they await the results of the litigation, petitions to Montana’s congressional delegation, and the delegation’s correspondence with the BIA, controversy continues. On February 12, 2021, Jon Tester (2021) wrote a letter to the new Acting Assistant Secretary of Indian Affairs Darryl LaCounte. It referenced a previous letter he had sent on October 30, 2020, about public safety on the Northern Cheyenne Reservation that had been ignored by the previous administration, despite their verbal commitments to improving communication with the Tribal Council, maintaining ten law enforcement officers on the reservation, and working with the tribe in good faith to proceed with the proposed 638 contract. Until that happens, the tribe must continue working with the BIA, although problems persist. On July 21, 2021, Northern Cheyenne President Donna Fisher (2021a, 2021b) wrote a letter to BIA Special Agent in Charge Lenora Nioce about the tribe’s resolution to remove BIA OJS Chief of Police Derris Waukazoo from the reservation, citing his disrespectful and unprofessional behavior towards the Tribal Council.

The tribe also depends on the federal government to provide jails. That problem, too, was exacerbated by the coronavirus pandemic, and further illustrates the problem of BIA shirking. The BIA decided to make arrests only for violent crimes (murder, rape, and serious assaults) soon after the pandemic began. According to the July 24, 2020, letter from Northern Cheyenne President Pena (2020) to Montana’s congressional delegation mentioned above, the 17-bed Lame Deer Adult Detention Facility had been the most overcrowded jail in the BIA system. The jail was closed in early 2019, over the tribe’s objections, following the opening of the Rocky Mountain Regional Detention Center (RMRDC), more than 60 miles away in Hardin. In April 2020, Deputy Bureau Director Charles Addington promised that the Lame Deer jail would be reopened partially in the very near future under the stipulations that it be a temporary holding facility for intoxicated individuals for a maximum of eight hours per incident. Pena stated that such limited use of the facility was inadequate and that the Northern Cheyenne tribe needed their own fully operational jail because arresting and detaining a suspect at the RMRDC required a 120-mile round trip by the arresting officer. In addition, she stated that since the Tribal Court and Indian Health Services hospital are in Lame Deer, adjudication and treatment of inmates would be much easier if the jail in Lame Deer were used. Furthermore, at the time the letter was written, the RMRDC was limiting its inmate population to only 100 beds even though the jail’s full capacity is 420 beds.

Senator Jon Tester (2020b) followed up by meeting with Tara Sweeney and Charles Addington on August 28, 2020, during which the latter two committed to meeting with President Peña and the Tribal Council to discuss next steps. Tester urged Sweeney and Addington to make the repairs necessary to reopen the Lame Deer jail as an eight-hour holding facility (rather than as a full-service facility as the tribe had requested). More than five months later, Tester (2021) wrote a letter to new Acting Assistant Secretary of Indian Affairs Darryl LaCounte again requesting that repairs to the jail be completed quickly.

On July 6, 2021, the Northern Cheyenne Tribe issued a press release noting that the BIA’s OJS had promised in August 2020 to reopen the jail by October 15, 2020, although at that point it was functioning at half capacity and continued to through 2021. While the tribe previously had participated in weekly meetings on the status of the jail’s reopening, the BIA OJS canceled the meetings, instead promising weekly reports to the tribe. However, when members of the Tribal Council toured the jail on June 15, 2021, they learned that it would have the capacity to hold only nine male and nine female inmates and, moreover, that the jail would indeed be downgraded to a temporary holding facility.

On December 29, 2021, Northern Cheyenne President Donna Fisher (2021b) wrote to BIA Special Agent in Charge Lenora Nioce. The letter addressed the fact that the Lame Deer jail had been converted into a facility to house juvenile offenders due to heating issues at the Youth Detention Facility in Busby, Montana, and questioned why the jail was able to house them for 24 h a day when the BIA’s OJS consistently had advised the tribe that the jail could hold adults only for eight hours. Of course, the fact that the Lame Dear jail was housing juvenile offenders meant that it could not also house adult offenders. Fisher therefore inquired whether the RMRDC would be expected to accommodate all people arrested for driving under the influence of alcohol or drugs during New Year’s Eve. She also asked why the youth detention facility’s administrator was assigned to the Lame Deer jail rather than dealing with the Busby facility’s problems.

The correspondence between the Northern Cheyenne and the federal government documents that the tribe has little control over how criminal justice facilities are used or managed. The lack of such control imposes tremendous costs on tribal citizens, who suffer from the insecurity associated with an understaffed police force having to travel more than 100 miles when an arrest is made because they have no local jail.

### Self-governance: military societies on the Northern Cheyenne Reservation

The account above captures the enforcement problems created by federal government actions. Here, we describe how military societies responded.

To understand the response, it is useful to first consider the features of Cheyenne military societies in greater detail. The four original warrior societies were the Fox, the Elk (or Elk Horn Scrapers, Bone Scraper, and others), the Shield Warrior Society (including its Buffalo Warriors branch), and the Bowstring (founded by Cheyenne warrior Owl Man–Owl Man’s bowstring). The fifth society was the Dog Warrior Society (Dog Men, called Dog Soldiers by whites), established by a visionary directive after legendary Cheyenne prophet Sweet Medicine’s departure; and a sixth society, the Contrary Warrior Society. Historically, each of those warrior societies comprised about 150 men and a chief.^4^

Leo Killsback’s (2020) account of Cheyenne military societies describes their key features and their historical relationship in the broader structure of Cheyenne tribal governance. Tribal governance of the Cheyenne Nation relied on shared responsibility and authority of several leaders and institutions—ten bands each led by four band chiefs who elected four principal chiefs to manage the entire Nation, thus creating a Council of Forty-four chiefs. This traditional government included four highly organized warrior societies who elected a number of headmen (usually four). Military societies and the council held each other accountable for poor decisions and outcomes. Military societies were legitimate because they were established long ago and their legacies were preserved in the tribe’s sacred history, as well as because of their organizational stability, reputation, membership, and governance. Warrior societies that lost credibility would not be assigned as many tasks.

Military societies are thus sacred institutions whose legitimacy also reflected their ability to contribute to the common good of tribal life. Against this cultural background, and the specific challenges posed by federal decisions during the coronavirus pandemic, it should not be surprising that the tribe called on these institutions in response to perceptions of lawlessness. The military societies never went away, and when lawlessness became more of a challenge, the Northern Cheyenne government knew they could call on these societies.

The response to the tribal government’s call for assistance was swift. The members of military societies, including the Elk Horn Scrapers, formed a camp. That camp had a security apparatus. They called their camp the People’s Camp and called their police force the People’s Camp Security. Its legitimacy was based on cultural institutions that predate European contact and which remain a significant aspect of current Cheyenne culture.

The military society had important roles in the pandemic. They initially ran reservation border checkpoints, stopped vehicles, and asked drivers from outside Montana to bypass the reservation; they then expanded their authority to include policing the tribe by imposing traditional punishments, including whippings with a chokecherry switch. One of the men, designated a “whipping man,” used the switch six times, all for intoxication, which he claimed was not a “beating”—it was, more accurately, described as corporal punishment, consistent with traditional rules of military societies. In other words, calling it a beating equivocates the legitimate punishment meted out by authorized society members with unjust, illegitimate physical violence. These additional public service functions reflected the perception that lawbreaking was on the rise during the pandemic though as the accounts above indicate, there was a perception that lawlessness on the reservation was an issue even before the pandemic as a result of lack of federal investment in providing law and order on the reservation.

Military societies thus evolved, as they have in the past (recall the evolution of military societies with diffusion of horses and with the rising threat from the US military starting circa 1850). Like before, there was an authorization from the tribal government—a call to action. There was also continuity of the public service motivation for participation in the activities of the military society. The People’s Camp and its members were volunteering their time; they were not paid employees of government, but privately contributing to the public good of security.

It was also governed by traditional rules, some of which were controversial. The society asked Rynalea Pena, the tribe’s president, not to participate because traditional values did not allow women to oversee the society’s activities (Hamby, 2020). Then-president Pena would later seek to disband the People’s Camp security, though as far as we can tell, the society continued to have a presence in the community even as the pandemic has become more manageable. Thus, a significant question, which we return to in the discussion section, is how the role of military societies will continue to evolve and their functions in public safety as the pandemic continues to wane.

Military societies were effective because they are accountable, legitimate and have the capacity to enforce the law. One might also ask whether there was something different about this particular reservation. Indeed, the Northern Cheyenne Reservation is far from any large cities. The relative isolation may have contributed more robust traditional institutions on the reservations. The location may also have placed a premium on self-reliance, thus making it more likely to return to traditional enforcement.

## Discussion

### Order without law

The authority of military societies has always been infused with tribal government and hence with “law.” They are not, in this regard, extralegal organizations as is often referred to in the literature on order without law. But from the perspective of even tribal law, they relied on the types of extralegal enforcement. Ellickson (1991) documented how ranchers settle disputes in contemporary Shasta County, California: ranchers would castrate the bulls of neighbors who persistently failed to abide by community norms. It was nowhere legal to do so, but since few bulls were castrated, it appears that it was an effective enough deterrent.

Among the Northern Cheyenne military societies, the chokecherry switch was a traditional method to punish individuals for transgressions of law. Though the military societies were authorized by the government, the use of corporal punishment was not specifically authorized by the tribal government, nor is it a part of any (legal) federal enforcement. But as we know from public choice analysis of punishment, the types of punishment may seem unusual or even morally repugnant, but may be considered legitimate and hence effective from the perspective of different cultures (Leeson, 2017). In the case of the Northern Cheyenne, both the use of the switch and exclusion of women from decision-making, while questionable by certain standards, are based in deeply held traditional beliefs. And if the accounts summarized above are accurate, they arguably are effective in addressing social needs.

### Institutional stickiness

One of the themes of Indian country is the top-down imposition of institutions. Boettke et al. (2008) categorize institutions as being imposed externally (through foreign intervention, colonialism, imperialism), internally by domestic governments, or as arising endogenously. Endogenous emergence is desirable from individuals’ points of view—if institutions persist, it can be inferred that they are preferred; Leeson (2020) argues that such institutions as efficient, in that the cost of changing them exceeds benefits. Endogenous institutions are desirable because they are based in practices, customs, and values of indigenous people. Their foundation is *metis*—local knowledge, resulting from local practice, including skills, cultures, norms, and conventions shaped by the experiences of individuals and which apply to interactions between people and the physical environment. Norms can be transmitted and evolve intergenerationally. But they are not easily replaced. Successful foreign intervention hinges on recognizing the importance of pre-existing local customs and institutions (Coyne, 2007).

The challenges confronting Indian country involve issues similar to those raised by the imposition of foreign-introduced institutions. The BIA was (and is) an alien, exogenous institution. The return of the Elk Horn Scrapers constituted the reemergence of indigenous institutions. This does not necessarily mean tribes do not need or want additional spending on policing services. To be sure, there is a need for resources on reservations. But institutional stickiness is relevant because there needs to be some constraints on even local governments. Fiscal federalism does not eliminate the problem of predatory policing as budgets are not the only source of such behavior (Skarbek, 2021). Military societies, and the belief in traditional policing as a way to improve policing, signifies the ongoing challenges posed by a legacy of exogenously imposed policing institutions, as well as institutional failures associated with policing at any level of government, including tribal government.

### Coproduction and criminal justice

Military societies can also be thought of as coproducers of public goods and services. Parks et al. (1981) observed that citizens are consumers and coproducers of public goods. Coproduction is the mix of activities in which citizens, communities, and public organizations work together to provide public goods and services. Historically, policing was coproduced—the Cheyenne and Crow, for example, had appointed warriors enforce the laws, and they worked together to ensure accountability. Historically, centralization of policing undermined this relationship.

Significantly, military societies are members of the tribes and have skin in the game in terms of living on the reservation. The fact that a good portion of their manpower is in the form of unpaid volunteers means they are intrinsically motivated in their task, unlike bureaucrats who primarily want to see their paychecks keep coming, which is not contingent on providing good public safety outcomes. One lesson is that volunteering can lead to effective provision; a second is that increased funding does not necessarily solve the problem of policing.

There remain substantial challenges to self-governance of policing. Federal policing is highly centralized; this centralization has been implicated in ongoing challenges on Indian reservations with high rates of crime, missing women and girls, and domestic violence on reservations, all of which exceed comparable off-reservation communities (Crepelle, 2021). These challenges are not solved through tribal policing contracts, whereby the tribes and federal government agree for the tribes to take over more policing responsibility. As Crepelle et al. (2022) showed, the complicated nature of federal laws of criminal jurisdiction mean that even tribal policing provides only partial sovereignty over criminal justice, thus undermining the success of decentralization of policing in improving public safety on reservations. Therefore, a conclusion from this research is that consolidation likely contributed to crime on reservations, and that federal rules governing criminal jurisdictions continue to undermine tribal ability to provide for criminal justice on reservations.

Recently, the Supreme Court’s decision in *Oklahoma v. Castro-Huerta* (2022) further eroded tribal autonomy over policing decisions on their reservations. In the case, tribes and former United States Attorneys argued granting states more authority over reservation crimes would lead to law enforcement shirking. Disregarding the perspective of those actually involved with Indian country safety, five Supreme Court Justices believed unilaterally imposing state police forces on tribal land was in the best of interests of the tribes. If *Castro-Huerta* leads to even more non-Indian law enforcement shirking and greater reliance on self-governing policing—despite tribal jurisdiction limitations—it may be tribes’ best hope for public safety.

## Conclusion

McChesney (1990) showed that there were large social costs when the federal government began defining and enforcing property rights on reservations. Our paper adds to the public choice analysis of federal enforcement, in this case, policing. Historically, the federalization of policing was the imposition of foreign institutions and supported by progressive ideology. The cost of progressive ideology is that it was premised on dismantling Indian institutions and replacing them with the federal government. It was inefficient: Indians had their own policing institutions, and with autonomy could have continued to police reservations. But political decisions are made not by what is best for society, but by what is best for political decision-makers. And in the case of policing on reservations, political interests, rather than what was best for Indians, governed changes in policing institutions on reservations.

We argued that criminal justice institutions require access, legitimacy and capacity. Among the Plains Indians, military societies historically served as the enforcers of rules of the tribes. These military societies were authorized by tribal government, and if they performed well in their duties, they received additional duties; if not, they lost out on opportunities to contribute to the tribe’s well-being. The reason why they were effective is because they were accessible to tribal citizens, they were legitimate, and they had capacity to enforce law. They were accessible because their members were part of the tribe. The military societies were legitimate because they emerged even before European contact, were appointed through processes that tribal members accepted, and because they were effective in providing order on reservations. They had capacity as well, as individuals appointed to these societies had skills and experience that made them effective in their role in criminal justice. Federal enforcement, in contrast, falls short on each of these dimensions: federal police were largely unaccountable to tribal governments, the imposition of federal criminal justice institutions has always had questionable legitimacy, and federal shirking means enforcement capacity is an issue.

Military societies were not a perfect system of criminal justice. Many tribes began to modernize tribal policing, moving away from traditional policing, even before the federal government imposed federal enforcement. The federal government interfered with this evolutionary process, imposing institutions of enforcement on reservations. The problems from that imposition continue to reverberate to this day. But what is most significant is comparing imperfect institutional alternatives, and from a comparative perspective, military societies provided an important foundation for indigenous evolution of policing institutions whereby modern tribal arrangements built upon military societies.

Fortunately, military societies remain a significant aspect of tribal cultures. Lear (2006) notes that a great challenge to the Plains Indians as the Indian Wars came to a conclusion was that warrior societies could not hunt buffalo as they once did. The warrior ideology continued in other ways. One reason the Northern Cheyenne case is interesting is because the military society performed similar functions as it did in the past. These societies are examples of self-governance: an all-volunteer organization provided policing services on the reservation. In Indian country, tribes have limited sentencing authority which inhibits their ability to effectively punish major crimes. Major crimes, as noted, are also a persistent challenge on reservations. Military societies might even be able to do an effective job contributing to investigations of major crimes such as rape or murder, though they are not themselves a source of formal adjudication. Since federal authority has proven so lacking, it may make sense to see how to better integrate military societies into additional aspects of policing on reservations.

Indeed, the coronavirus pandemic provides something of a natural experiment to see whether societies can reemerge, and if so, whether they can positively contribute to improving public safety in Indian country. In one of the most significant studies of policing on reservations, Wakeling et al. (2001) found that tribal policing typically works better than federal or state policing, and so tribes should have every opportunity to move in that direction if they so desire. In their interviews at the Fort Berthold Reservation in west-central North Dakota, several of their interviewees brought up the Black Mouth Society, an association in which older, courageous males played a central role in maintaining order during pre and early reservation life, where behavior was enforced simply by the threat that people would tell the Black Mouths. If the Northern Cheyenne is any guide, it suggests that the Black Mouths may not be a distant memory.

## Footnotes

1 This power over non-Indians was established in the Treaty between the United States of America and the Northern Cheyenne and Northern Arapahoe Tribes of Indians of 1868, which states, “If bad men among the whites, or among other people subject to the authority of the United States shall commit any wrong upon the person or property of the Indians, the United States will, upon proof made to the agent and forwarded to the Commissioner of Indian Affairs at Washington City, proceed at once to cause the offender to be arrested and punished according to the laws of the United States, and also re-imburse the injured person for the loss sustained.”

2 For a public choice perspective on gift giving and its role in tribal governance, see Johnsen (2022), who explains how gifting salmon and valuable salmon runs contributed to political order and economic well-being among Pacific Northwest Indians prior to European contact.

3 While the majority in *Oklahoma v. Castro-Huerta* clearly declared that the decision applies throughout the United States, Justice Gorsuch’s dissent notes that the majority’s reasoning limits the case to eastern Oklahoma. Thus, state law enforcement arguably still lacks jurisdiction over Indians on the Northern Cheyenne Reservation.

4 Dorsey (1905) provides a history of the Cheyenne military society.

## References

[CR1] Aadland, C. (2020, December 18). Tribe sues feds over policing. *Montana Free Press*. https://montanafreepress.org/2020/12/18/northern-cheyenne-sues-feds-over-policing/.

[CR2] Aligica PD, Boettke PJ, Tarko V (2019). Public governance and the classical-liberal perspective: Political economy foundations.

[CR3] Allen DW, Barzel Y (2011). The evolution of criminal law and police during the pre-modern era. The Journal of Law, Economics, and Organization.

[CR4] Anderson TL, McChesney FS (1994). Raid or trade? An economic model of Indian-White relations. Journal of Law and Economics.

[CR5] Anderson, T. L., & Parker, D. P. (2022). Culture, sovereignty, and the rule of law: Lessons from Indian country. *Public Choice*.

[CR6] Associated Press. (2022). Northern Cheyenne Tribal Council Votes to Remove President. *US News & World Report*. https://ww.usnews.com/news/best-states/montana/articles/2022-02-03/northern-cheyenne-tribal-council-votes-to-remove-president. Accessed 7 August 2022.

[CR7] Benson BL (1994). Are public goods really common pools? Considerations of the evolution of policing and highways in England. Economic Inquiry.

[CR8] Blackburn BL (1980). From blood revenge to the Lighthorsemen: Evolution of law enforcement institutions among the five civilized tribes to 1861. American Indian Law Review.

[CR9] Boettke PJ, Coyne CJ, Leeson PT (2008). Institutional stickiness and the new development economics. American Journal of Economics and Sociology.

[CR10] Boettke PJ, Lemke JS, Palagashvili L (2016). Re-evaluating community policing in a polycentric system. Journal of Institutional Economics.

[CR11] Candela RA, Geloso VJ (2018). The lightship in economics. Public Choice.

[CR12] Clark JR, Powell B (2019). The ‘minimal’ state reconsidered: Governance on the margin. The Review of Austrian Economics.

[CR13] Cowen T, Sutter D (2005). Conflict, cooperation and competition in anarchy. The Review of Austrian Economics.

[CR14] Coyne, C. J. (2007). *After War: The Political Economy of Exporting Democracy*. Palo Alto, CA: Stanford Economics and Finance.

[CR15] Crepelle A (2016). Concealed carry to reduce sexual violence against American Indian women. Kansas Journal of Law and Public Policy.

[CR16] Crepelle A (2021). The law and economics of crime in Indian country: Why things are so bad. Georgetown Law Journal.

[CR17] Crepelle, A., Fegley, T., Murtazashvili, I., & Murtazashvili, J. B. (2022). Community policing on American Indian reservations: a preliminary investigation. *Journal of Institutional Economics*, pp. 1–18.

[CR18] Daines, S. (2020). Letter to William Barr and David Bernhardt. https://tinyurl.com/2p8x2uvh.

[CR19] Dorsey, G. A. (1905). *The Cheyenne* (Vol. 9). Rio Grande Press.

[CR20] Ellickson RC (1991). Order without law: How neighbors settle disputes.

[CR21] Fisher, D. (2021a). Letter to Lenora Nioce. https://tinyurl.com/4349ykhw.

[CR22] Fisher, D. (2021b). Letter to Lenora Nioce. https://tinyurl.com/cdm766sv.

[CR23] Greif A (2006). History lessons: The birth of impersonal exchange: The community responsibility system and impartial justice. Journal of Economic Perspectives.

[CR24] Hadfield GK, Weingast BR (2012). What is law? A coordination model of the characteristics of legal order. Journal of Legal Analysis.

[CR25] Hagan, W. T. (1966). *Indian police and judges: Experiments in acculturation and control*. Yale University Press.

[CR26] Hamby, P. (2020, July 23). Northern Cheyenne tribal members policing reservation.

[CR27] Holcombe, R. G. (2019). Progressive Democracy: the ideology of the modern predatory state. *Public Choice*, pp. 1–15.

[CR28] Humphrey ND (1942). Police and tribal welfare in Plains Indian cultures. Journal of Criminal Law and Criminology.

[CR29] Johnsen, D. B. (2022). Potlatch Economy: Reciprocity at Work. *Public Choice*.

[CR30] Jones OL (1966). The origins of the Navajo Indian Police 1872–1873. Arizona and the West.

[CR31] Karr SM (1998). Now we have forgotten the old indian law: Choctaw culture and the evolution of corporal punishment. American Indian Law Review.

[CR32] Killsback, L. K. (2020). *A sacred people: Indigenous governance, traditional leadership, and the warriors of the Cheyenne Nation*. Texas Tech University Press.

[CR33] Lear, J. (2006). *Radical hope: Ethics in the face of cultural devastation*. Harvard University Press.

[CR34] Leeson PT (2007). Anarchy, monopoly, and predation. Journal of Institutional and Theoretical Economics.

[CR35] Leeson PT (2017). WTF?!: An economic tour of the weird.

[CR36] Leeson PT (2020). Logic is a harsh mistress: Welfare economics for economists. Journal of Institutional Economics.

[CR37] Leeson PT, Coyne CJ (2012). Sassywood. Journal of Comparative Economics.

[CR38] Leeson, P. T., & Suarez, P. A. (2015). Superstition and self-governance. In *New Thinking in Austrian Political Economy* (pp. 47–66). Emerald Group Publishing Limited.

[CR39] Luna-Firebaugh E (2007). Tribal policing: Asserting sovereignty, seeking justice.

[CR40] McChesney FS (1990). Government as definer of property rights: Indian lands, ethnic externalities, and bureaucratic budgets. Journal of Legal Studies.

[CR41] Meadows WC (2002). Kiowa, Apache, and Comanche military societies: Enduring veterans, 1800 to the present.

[CR42] Northern Cheyenne Tribe. (2020). Northern Cheyenne Tribe Sues BIA for Failure to Address Public Safety. https://tinyurl.com/bdhrx55k

[CR43] Olson M (1993). Dictatorship, democracy, and development. The American Political Science Review.

[CR44] Parks RB, Baker PC, Kiser L, Oakerson R, Ostrom E, Ostrom V (1981). Consumers as coproducers of public services: Some economic and institutional considerations. Policy Studies Journal.

[CR45] Pena, R. (2020). Letter to Steve Daines, Jon Tester, and Greg Gianforte. https://tinyurl.com/bdfchsbz.

[CR46] Skarbek EC (2021). Fiscal equivlalence: Principle and predation in the public administration of justice. Social Philosophy and Policy.

[CR47] Surprenant, C. W., & Brennan, J. (2019). *Injustice for All: How Financial Incentives Corrupted and Can Fix the US Criminal Justice System*. Routledge.

[CR48] Tester, J. (2019). Letter to Christopher Wray. https://tinyurl.com/2p8tz6vr.

[CR49] Tester, J. (2020a). Letter to Tara Sweeney and Christopher Wray. https://tinyurl.com/mu8b5267.

[CR50] Tester, J. (2020b). Letter to Tara Sweeney and Charles Addington. https://tinyurl.com/2p8kw4z7.

[CR51] Tester, J. (2021). Letter to Darryl LaCounte. https://tinyurl.com/7b6v9e6s.

[CR52] Tyler TR (2003). Procedural justice, legitimacy, and the effective rule of law. Crime and Justice.

[CR53] Umbeck J (1977). The California gold rush: A study of emerging property rights. Explorations in Economic History.

[CR54] Wakeling, S., Jorgensen, M., Michaelson, S., & Begay, M. (2001). Policing on American Indian Reservations. *National Institute of Justice, US Department of Justice, Washington, DC.*

